# Successful Management With Continuous Negative Abdominal Pressure Therapy in a Severely Obese Patient With Inhalation Burn-Induced Severe Respiratory Failure Requiring Veno-Venous Extracorporeal Membrane Oxygenation: A Case Report

**DOI:** 10.7759/cureus.57436

**Published:** 2024-04-02

**Authors:** Naoki Kawahara, Hiroki Matsui, Koji Morishita

**Affiliations:** 1 Trauma and Acute Care Surgery, Tokyo Medical and Dental University Hospital, Tokyo, JPN

**Keywords:** atelectasis, respiratory failure, inhalation burns, severe obesity, continuous negative abdominal pressure

## Abstract

Continuous negative abdominal pressure (CNAP) therapy effectively provides respiratory support in patients with respiratory failure and severe obesity; however, its use in clinical practice remains limited. In this case, we report a significant improvement in the respiratory condition of a patient with severe obesity and inhalation burns following the application of CNAP in addition to venovenous extracorporeal membrane oxygenation (V-V ECMO) and mechanical ventilation. The patient was able to wean off these devices successfully. This case highlights the potential of CNAP therapy as an adjunct treatment for severe respiratory failure, particularly in obese patients for whom conventional interventions are insufficient.

## Introduction

Inhalation burns can occur owing to the inhalation of flames or hot smoke during fires. While inhalation burns can lead to laryngeal edema, resulting in upper airway obstruction, they can also cause lower airway obstruction due to edema, mucosal sloughing, and increased secretion, leading to respiratory failure [1]. Obesity also affects respiratory function by decreasing compliance, reducing capacity, increasing airway resistance, and weakening the respiratory muscles, partly because of elevated intra-abdominal pressure [2]. Continuous negative abdominal pressure (CNAP) therapy, which externally lowers intra-abdominal pressure by applying continuous negative pressure, has been proposed as a treatment modality [3].

Here, we report a case of severe respiratory failure in a severely obese patient with inhalation burns, requiring venovenous extracorporeal membrane oxygenation (V-V ECMO), and the successful use of CNAP therapy with an RTX respirator (United Hayek Industries (Manufacturing) Ltd., London, UK) to improve respiratory status.

## Case presentation

A 67-year-old male with a history of hypertension, diabetes, and renal cancer was rescued from a home fire. The patient had a height of 162.0cm, a weight of 129.3kg, and a BMI of 49.3 kg/m^2^. On arrival at our center, vital signs were stable except for tachycardia. Soot was adherent around his mouth, and there was a superficial burn of 1.0% of the total body surface area on his face and forehead. Neck auscultation revealed stridor. Arterial blood gas analysis revealed a high carboxyhemoglobin (CO-Hb) level of 25.6%. On laryngeal fiberoptic examination, the upper oropharynx exhibited signs of swelling in the soft palate mucosa, epiglottis, and arytenoid region, with evidence of soot deposition throughout the pharyngolaryngeal area. Tracheal intubation was performed due to the prediction of further progression of upper airway obstruction based on fiber optic findings. Based on elevated CO-Hb levels, we diagnosed the patient with carbon monoxide poisoning and administered hyperbaric oxygen therapy. The patient was subsequently admitted to the intensive care unit.

**Figure 1:**
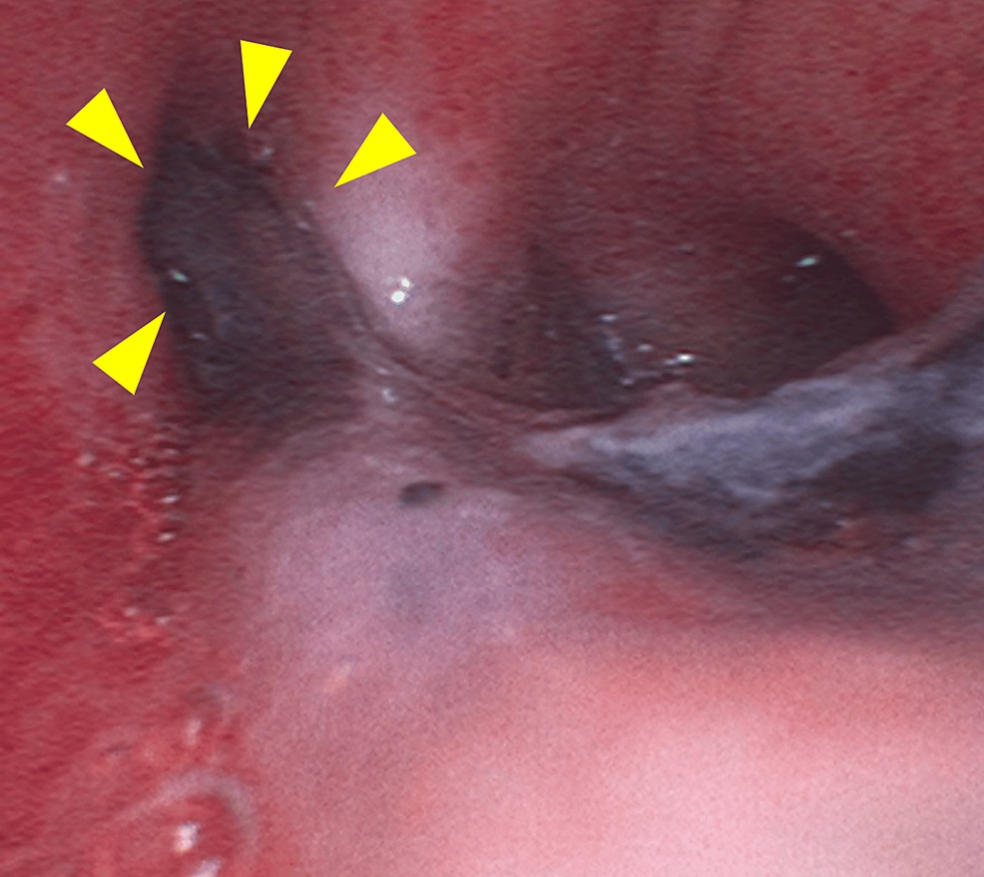
Bronchoscopy findings on the 2nd hospital day. The image shows bronchial obstruction due to mucus (yellow arrows) and bronchial mucosal edema.

**Figure 2:**
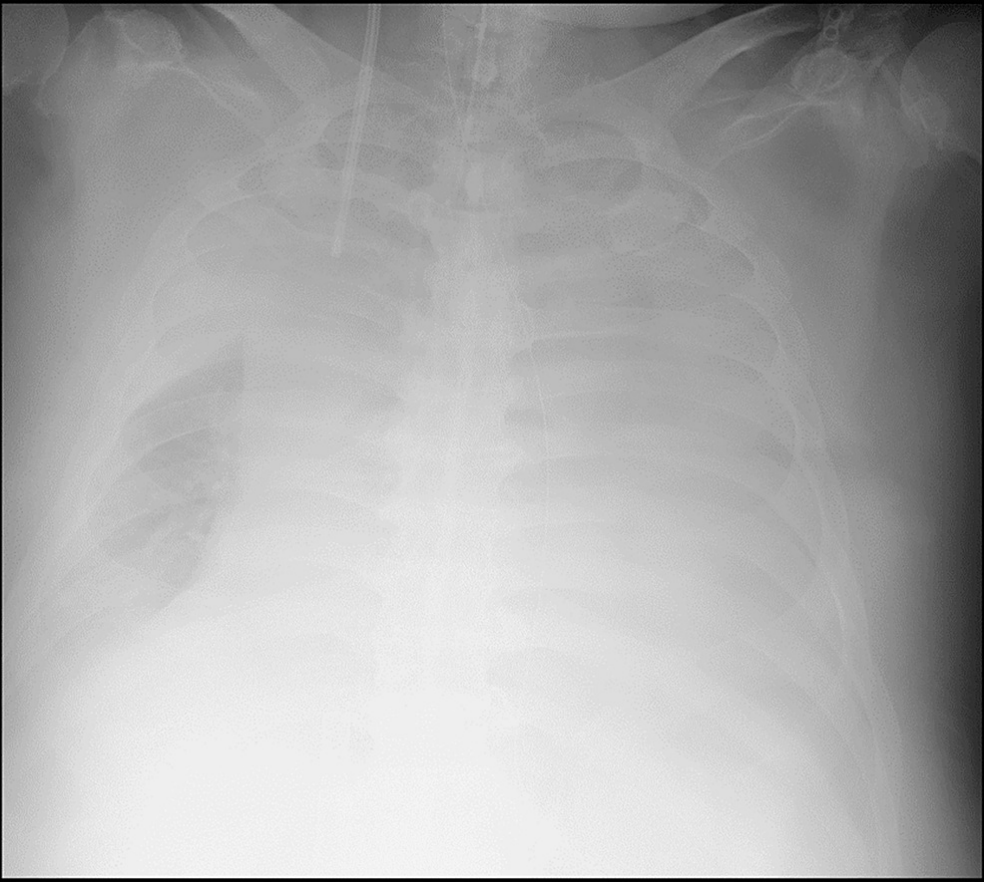
Chest X-ray on the 5th hospital day (before CNAP therapy). The image shows extensive bilateral atelectasis. CNAP: Continuous negative abdominal pressure

The initial PaO_2_/FiO_2_ (P/F) ratio upon admission was 314mmHg. On the second hospital day, the respiratory condition became poor in both oxygenation and ventilation, with ventilator settings of PEEP 10cmH_2_O and driving pressure 20cmH_2_O, resulting in a P/F ratio of 150-160 mmHg, minute volume of approximately 8.0L and pCO_2_ 70-75 mmHg. Maintaining respiratory function with mechanical ventilation alone became difficult, prompting the initiation of V-V ECMO. After V-V ECMO initiation, we changed the ventilator settings of PEEP 12cmH_2_O and driving pressure 5cmH_2_O for resting lung. On the 3rd hospital day, the left lung developed atelectasis, and bronchoscopy revealed an edematous lower airway mucosa with mucous plugging (Figure 1). On the fifth hospital day, chest radiography revealed extensive bilateral atelectasis (Figure 2). Despite efforts directed towards ventilator management focused on resting lung ventilation and bronchoscopy-assisted suctioning, improvement in respiratory function remained minimal, and the tidal volume gradually decreased over time, reaching approximately 0.5-1.5L/min. Repositioning the patient to right lateral recumbency resulted in hypotension, which was attributed to the inferior vena cava (IVC) compression by the abdominal fat, leading to reduced venous return.

**Figure 3:**
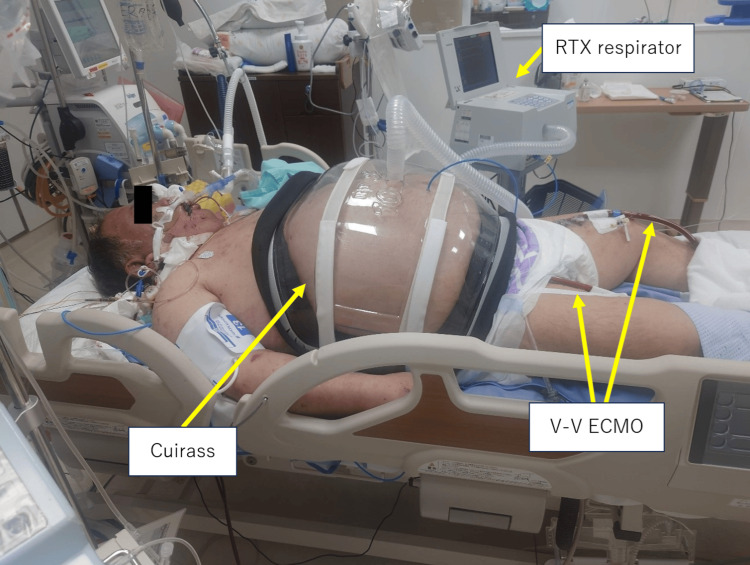
The patient is undergoing CNAP therapy with a cuirass applied to the abdomen. CNAP: Continuous negative abdominal pressure; V-V-ECMO: venovenous extracorporeal membrane oxygenation

**Figure 4:**
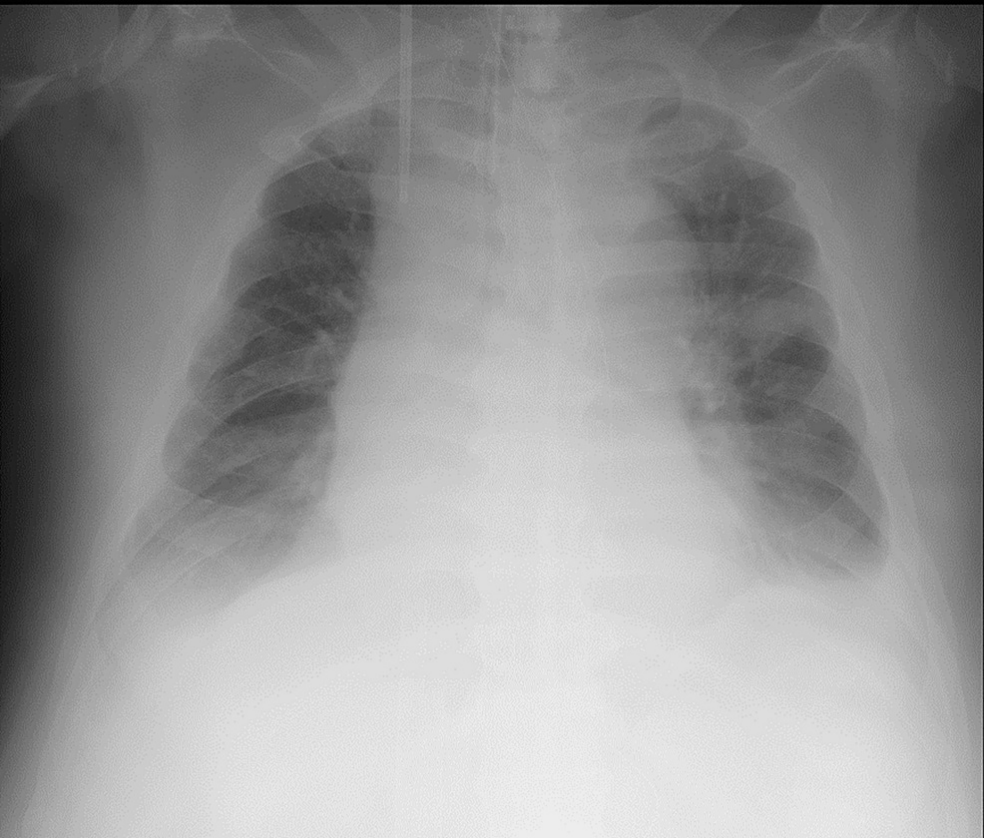
Chest X-ray on the 13th hospital day (after CNAP therapy) The image shows improved atelectasis. CNAP: Continuous negative abdominal pressure

Consequently, altering the patient's position for sputum drainage proved difficult. Despite repeated bronchoscopy-assisted suction, extensive atelectasis persisted. Considering that elevated intra-abdominal pressure due to severe obesity contributes to respiratory function decline, CNAP therapy with an RTX respirator was initiated on the 12th day of hospitalization (Figure 3). We used the Continuous Negative mode and applied 20 cmH_2_O negative pressure to the patient's abdomen using the RTX respirator's cuirass interface. CNAP therapy was administered for approximately six hours only during the daytime to prevent pressure ulcers on the skin at the cuirass interface. Atelectasis on chest radiography showed remarkable improvement from the 13 to 16th hospital day (Figure 4). On the 17th day of hospitalization, the patient's respiratory status improved, leading to weaning off the V-V ECMO. Although respiratory status gradually improved, early liberation from mechanical ventilation was challenging because of respiratory muscle fatigue resulting from disuse. Hence, we performed a tracheostomy on the 20th day of hospitalization. Continued rehabilitation gradually improved the disuse syndrome, and mechanical ventilation was weaned off on the 60th hospital day. Although consciousness impairment presumedly caused by carbon monoxide poisoning persisted, gradual improvement was observed. Thus, the level of consciousness improved, and the patient became able to obey commands on the 61st hospital day. The patient was transferred for rehabilitation purposes on the 62nd hospital day.

## Discussion

To our knowledge, this is the first case report of CNAP therapy for atelectasis induced by inhalation burns. In this case, we effectively managed severe respiratory failure precipitated by inhalation burns in a severely obese patient using V-V ECMO for respiratory support and CNAP therapy with an RTX respirator, ultimately leading to successful weaning from ECMO and mechanical ventilation. In this case, atelectasis primarily caused respiratory failure without accompanying circulatory failure, making respiratory support with V-V ECMO effective.

The patient had a body mass index (BMI) of approximately 50 kg/m^2^, indicating severe obesity. Obesity is associated with elevated intra-abdominal pressure [4], predisposition to diaphragmatic elevation, and increased susceptibility to alveolar collapse, which leads to atelectasis [5,6]. Additionally, the patient's condition was compounded by lower airway edema and increased secretions due to inhalation burns, further escalating atelectasis risk [1]. Initially, respiratory support was maintained using V-V ECMO and suctioning while awaiting temporal improvement of airway burns, expecting atelectasis resolution. However, limited improvement resulted in the initiation of CNAP therapy in the abdominal cavity using an RTX respirator to reduce intra-abdominal pressure. Subsequently, we observed significant improvements in atelectasis and respiratory status. CNAP therapy acts as an adjunct to positive end-expiratory pressure [7] and has reportedly improved atelectasis in animal models but with limited reported effect in humans. In this case, CNAP therapy likely suppressed diaphragmatic elevation, improved lung compliance, and resolved the atelectasis. In patients with severe obesity and atelectasis, CNAP therapy may be an effective adjunct to open-lung strategies using mechanical ventilation.

Computed tomography (CT) revealed predominantly dorsal atelectasis. In cases of posterior-dominant atelectasis, prone positioning therapy could be effective because it may improve ventilation in the dorsal lung regions and facilitate the movement of gas to the lower lung regions using gravity, improving posterior-dominant atelectasis [8]. Furthermore, the prone position promotes the opening of the dorsal lung regions and aids in the clearance of secretions [8,9]. However, changing to prone positioning carries risks, such as hypotension and the potential for device dislodgement accidents [10]. Additionally, staff efforts are required to change positions, particularly in overweight patients. In this patient, a decrease in blood pressure was observed when placed in the right lateral decubitus position, attributed to compression of the inferior vena cava by abdominal fat. While the patient could assume the left lateral decubitus position without significant difficulty, we refrained from placing the patient in the prone position due to concerns regarding hemodynamic instability. CNAP therapy with an RTX respirator can be performed in the supine position, making it a vital option for patients with respiratory failure who are at risk of complications due to positional changes.

## Conclusions

Herein, we report a case of CNAP therapy that effectively resolved atelectasis in a severely obese patient with extensive burns. CNAP therapy is minimally invasive and easily administered. In severely obese patients, it may serve as a useful adjunct to open-lung strategies using mechanical ventilation, particularly in cases where elevated intra-abdominal pressure and difficulty in positional changes pose challenges.
